# Definitions, signs, and symptoms of constipation in people with severe or profound intellectual disabilities: A systematic review

**DOI:** 10.1016/j.heliyon.2022.e09479

**Published:** 2022-05-20

**Authors:** Marjolijn C. Wagenaar, Annette A.J. van der Putten, Johanna G. Douma, Cees P. van der Schans, Aly Waninge

**Affiliations:** a Research Group Healthy Ageing, Allied Health Care and Nursing, Hanze University of Applied Sciences Groningen, Groningen, the Netherlands; b Department of Inclusive and Special Needs Education, University of Groningen, Groningen, the Netherlands; c Department Health Psychology, University Medical Centre Groningen, Groningen, the Netherlands; d Department Rehabilitation Medicine, University Medical Centre Groningen, Groningen, the Netherlands

**Keywords:** Intellectual disabilities, Severe or profound, Constipation, Definition, Signs and symptoms, Systematic review

## Abstract

**Background:**

It is difficult to diagnose constipation for people with severe or profound intellectual disabilities. Definitions for this are ambiguous, and the symptoms and signs are often unnoticed. The aim of this study is to identify clear definitions of constipation for people with different levels of intellectual disabilities and to identify signs and symptoms.

**Method:**

Guided by the PRISMA statement, a systematic review of the literature was conducted within electronic databases MEDLINE, Embase, CINAHL, Cochrane, and PsycINFO. Definitions, signs, and symptoms were extracted and the quality of definitions was assessed.

**Results:**

In total, 24 studies were included. Quality of definitions ranged from poor to good quality. Standard and referenced definitions were used in ten studies, a self-composed definition was employed in eleven studies; and three studies did not refer to a source of the definition. The self-composed definitions had not been evaluated after being used for the target group, and no scientific substantiation was available. A broad range of signs and symptoms were described.

**Conclusions:**

No substantiated definition has been ascertained for constipation for people with severe or profound intellectual disabilities. Further research will be necessary to identify which signs and symptoms are important for defining constipation in this target group.

## 1 Introduction

Constipation is present in approximately 10–20% of the Dutch population (Enders, 2015) which is comparable with figures from international studies (Peppas et al., 2008). Constipation is not a disease but a collection of symptoms that occurs at varying degrees (Smout, 2001). Moreover, it is a complex condition which usually occurs with a multitude of different reasons, such as inactivity, inadequate or enteral nutrition, anti-epileptic drugs use (Murata et al., 2017). Constipation is defined by bowel symptoms of “difficult or infrequent passage of stool, hardness of stool, or a feeling of incomplete evacuation” (Bharucha et al., 2013). In persons with an intellectual disability, prevalence between 27% (McCarron et al., 2013) and 43.3% (Hermans and Evenhuis, 2014) were found based on self and/or caregiver reports, or laxative use respectively. Especially in those with a profound intellectual disability, constipation is common (Robertson et al., 2018). Also, according to support plans and medical records, 94% of people with a profound intellectual and multiple disability experience constipation (Van Timmeren et al., 2016). Moreover, digestive system disease prevalence, was found to be 2.5 times higher in people with intellectual disability compared to controls without intellectual disability (Patja et al., 2001). Medical problems that can arise due to chronic constipation are rectal prolapse, diverticula of colon, intestinal obstruction, megacolon, and hemorrhoids (Van Winckel et al., 1999). Also, it has been observed that a higher frequency of convulsions in persons with epilepsy may be related to constipation (Bragg et al., 1994). In fact, constipation can be a serious problem resulting in fatal intestinal obstruction due to missed clinical symptoms (Jancar and Speller, 1994; Patja et al., 2001), and these may cause death among individuals with severe or profound intellectual disabilities (Cvetković et al., 2019).

Persons with intellectual disabilities have significant limitations in both intellectual functioning and adaptive behavior as expressed in their conceptual, social, and practical skills (Schalock et al., 2021). People with severe or profound intellectual disabilities need support in a lot of domains of functioning, for example regarding activities for daily living, language, motor skills, and sensory functioning (Nakken and Vlaskamp, 2007; Schalock et al., 2021). More specifically, also for signaling and identifying constipation, these persons are dependent on their primary caregivers i.e. their family, or direct support persons (Nakken and Vlaskamp, 2007). People with severe or profound intellectual disabilities namely cannot verbally tell about the presence of a physical health problem, but they communicate in a different way compared to others without disabilities about physical discomfort for example by presenting a change in mood or behavior (Nakken and Vlaskamp, 2007). And that while, people with more severe intellectual disabilities are more at risk for physical health problems such as epilepsy, motor problems, visual impairment, and constipation (Van Timmeren et al., 2017). Although constipation is often diagnosed in people with severe or profound intellectual disabilities, it is not clear how this diagnosis has been established. Moreover, there is no widely accepted definition for constipation for this population (Veugelers et al., 2010).

Not every person shows the same signs and symptoms when experiencing constipation (Smout, 2001). There are six criteria that can be investigated in faeces: form; consistency; frequency; colour; odor, and quantity. However, these six criteria only indicate something about the faeces and how it appears; they do not provide enough information to diagnose constipation (Bulechek et al., 2016). The most commonly used clinical definition of constipation is composed by the Rome Diagnostic Criteria (further *Rome criteria*) in which the many different forms of expression are represented (Herz et al., 1996; Longstreth et al., 2006). The Rome criteria (Sobrado et al., 2018) - consisting of medical history and physical examination - are the gold standard for diagnosing constipation for the general population (Gulati et al., 2018). However, the Rome criteria may have limitations for the use in persons with severe or profound intellectual disabilities (Coleman and Spurling, 2010). One of the main difficulties in diagnosing constipation in people with severe or profound intellectual disabilities, is that the clinician needs a reliable medical history which cannot be reported verbally by themselves. Also, some questions regarding symptoms cannot simply be answered by others, for example, the feeling of incomplete defecation or of anorectal obstruction or blockage. Furthermore, symptoms such as nausea and abdominal pain are complex to describe and/or communicate for children and adults with severe or profound intellectual disabilities (Koppen et al., 2017). In addition, one of the Rome criteria is ‘bowel movement less than three times a week’; however, a new insight is that the frequency of bowel movement in children with constipation can be even higher than in children without it (Gulati et al., 2018). In accordance with this insight, other authors state that it is more important to investigate deviation from a person's normal frequency of bowel movement and how much effort it takes to relieve (Enders, 2015). It is not clear which definitions are currently used to describe constipation in persons with severe or profound intellectual disabilities.

In summary, considering the high risks of constipation in people with severe or profound intellectual disabilities, it is important to properly diagnose it. However, the target group is dependent on their environment to recognize constipation in time (Coleman and Spurling, 2010) and, for healthcare professionals, it is often difficult to properly interpret the communication of these individuals. Moreover, because constipation can express itself in different forms, it is even more difficult for the environment of people with severe or profound intellectual disabilities to recognize their signals as constipation. Due to the communication difficulties, the vagueness of the symptoms, and the presence of other (medical) priorities, constipation is often overlooked by caregivers and clinicians (Marsh and Sweeney, 2008). The Rome criteria commonly used as a definition for constipation, may not be applicable to people with severe or profound intellectual disabilities (Bulechek et al., 2016). In addition, it is not clear which guidelines physicians currently use for diagnosing constipation in this group (Veugelers et al., 2010), and neither is it clear which definitions are used for describing constipation for them. Therefore, an overview of definitions as well as signs and symptoms that can be used for diagnosing constipation in persons with severe or profound intellectual disabilities is needed. This study focuses on studies containing a definition of constipation for people with intellectual disabilities in general because it is expected that there are only a small number of publications that specifically focus on constipation of people with severe or profound intellectual disabilities. The aim of this systematic review is to identify definitions of constipation including signs and symptoms in people with different levels of intellectual disabilities.

## 2 Methods

### 2.1 Design

The Preferred Reporting Items for Systematic Reviews and Meta-Analysis (PRISMA) statement, a guideline for reporting items of systematic reviews, was used when conducting and reporting this systematic review (Moher et al., 2009). After including the papers, definitions of constipation were identified, extracted, and assessed regarding the quality.

### 2.2 Search strategy

A search strategy (see Appendix A) was developed and employed to retrieve papers from five electronic databases: MEDLINE, Embase, CINAHL, Cochrane and PsycINFO. This search was conducted in February and March 2019. In order to provide an extensive overview of current literature, the search included broad terms related to people with an intellectual disability and the definition or diagnosis of constipation.

### 2.3 Selection of studies

#### 2.3.1 Inclusion criteria

To be included in the review, studies were required to meet the following inclusion criteria: only peer-reviewed journal papers published within the last 20 years (between January 1998 and December 2018) and written in English were eligible for inclusion. Furthermore, participants in the studies were required to be adults and/or children with intellectual disabilities. Studies needed to describe how constipation was defined, operationalized, or diagnosed.

#### 2.3.2 Exclusion criteria

Excluded were papers that were not peer reviewed or those of which the peer review status was unclear. Any study that did not describe a definition of constipation, and reviews, letters, commentaries, editorials, meeting or conference abstracts were excluded. Also, studies in which conditions about intellectual disability could not necessarily be assumed or when results were not disaggregated for people with an intellectual disability were excluded. In addition, studies about diseases, disorders, or syndromes with a different pathology that causes constipation, for example, congenital anal web, bowel obstruction caused by pica, coeliac disease, Hirschsprung disease, Crohn disease, and studies based on newborn infants were excluded.

#### 2.3.3 Screening process

In the first stage of the selection process, 10% of the title and abstract screening was conducted by two authors (MW and AW) resulting in 100% agreement; the remaining 90% of titles and abstracts were screened by one author (MW) whereby dubiety was resolved in consultation (MW with AW). Reading the full-text papers and completing an inclusion checklist were completed by two authors (MW and GD), and disagreements were resolved by a consensus discussion with two authors (MW and GD) and, if needed, with a third author (AW). When relevant information was missing, the present authors emailed the corresponding author.

#### 2.3.4 Assessment of the quality of the definition of constipation

The quality of the definition for constipation in people with (severe or profound) intellectual disabilities was assessed with the checklist for text and opinion critical appraisal of topic (CAT) (McArthur et al., 2015). The checklist was operationalized by two authors (MW and GD) with the operationalization checked by the other authors. This operationalization was necessary in order to be able to judge the quality of the definitions as there is no checklist that only focuses on them. Quality indicators of the description of the definition within the selected articles were the theoretical basis and focus on the target group. The following six criteria were scored: *1. The source of the definition was clearly identified by author; 2. At least one of the authors has standing in the field of expertise; 3. Interests of the population was a central focus of the definition; 4. Stated definition was the result of an analytical process, and there was logic in the argumentation expressed; 5. Reference to the extant literature (more than one article) and a non-biased representation were described; 6. Incongruence with the literature/sources was logically defended* (see Appendix B for the operationalized list based on (McArthur et al., 2015) with scores: yes (one point); no (zero points); unclear (zero points); not applicable (zero points)). A higher score on this checklist is indicative of a better quality of the definition (range: 0–6).

The quality assessment was conducted by two authors independently (MW and GD) for the included papers. The first author (MW) assessed all definitions, and the other author (GD) 25% of the studies. After consultation, there was 100% consensus about the assessments.

### 2.4 Data procedure

The following information of the included studies was described: name of the first author, year of publication, number of participants, IQ or a description of the level of intelligence of the participants, age, gender, topic of the study, country, study type, source of the definition used to describe constipation, and the score on the adjusted version of the CAT (McArthur et al., 2015).

Signs and symptoms of the definitions of constipation were extracted from the included papers. This was performed by one author (MW) whereby dubiety was resolved in consultation (with AW). The data related to the definitions for constipation used in the papers were shown as signs and symptoms. The extracted data were organized firstly by the scores of the quality assessment, and secondly by the frequency of the signs and symptoms that were found.

## 3 Results

**Figure 1:**
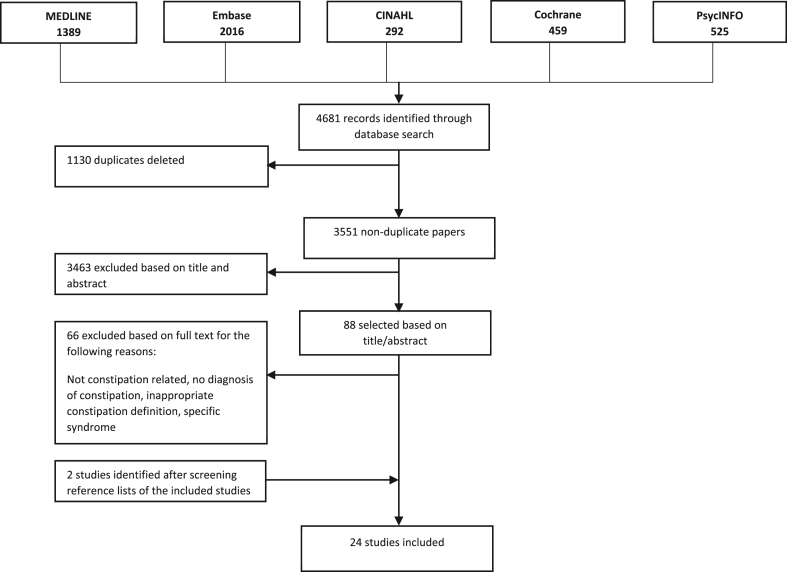
Flow diagram of selection process.

Figure 1 outlines the study selection in a flow diagram. A total of 24 studies satisfied the inclusion criteria.

Table 1 provides an overview of the 24 included studies. Of these studies, 16 were conducted between 2010 and 2019. The number of participants varied between one and 2283. There were 10 studies focusing on only children younger than 21 years; six studies included only adults; six studies included both children and adults; and of two studies age was not specified. The IQ range in the studies varied between <20 and 75 points. Participants with an IQ between 50 and <20 points were included in 12 studies that were conducted. There were five studies that did not specify the IQ of the participants and used the term ‘intellectual disabilities’. In 19 studies, more than half of the participants were male. In five studies, the ratio between men and women was unknown. There were 14 cross-sectional studies that primarily focused on prevalence of constipation and/or other gastro-intestinal symptoms in people with intellectual disabilities; the other ten studies were either comparative (1), prospective (1) or intervention studies, such as case control (4) or double-blind studies (2). Seventeen studies took place in Europe with most of them being conducted in the United Kingdom (six studies) and the Netherlands (five studies).

**Table 1:** Overview of included studies.

First author & year	Number of participants, IQ (in points), age (years), gender (%male), topic, country	Study type	Source of the definition	CAT for Text and Opinion Papers (McArthur et al., 2015)						
				1. Source	2. Expertise	3. Population	4. Analytical process	5. Reference literature	6. Incongruence defended	Total
1. Böhmer et al. (2001)	*n*: 215	Cross-sectional study	Harari et al., 1996	Y	Y	Y	N	Y	N	4
	IQ: <50									
	Age: range 6–80									
	Male: 60.0 %									
	Topic: Prevalence of constipation in people with ID living in a residential home									
	Country: The Netherlands									
2. de CarvalhoMrad et al. (2014)	*n*: 84	Cross-sectional study	Rasquin et al., 2006	Y	Y	N	N	N	N	2
	IQ: ID									
	Age: range 4–30									
	Male: 33.3%									
	Topic: Prevalence of lower urinary tract symptoms in individuals with DS									
	Country: Brazil									
3. de Carvalho Mrad et al. (2018)	*n*: 204; 93 DS	Case-control study	Rasquin et al., 2006	Y	Y	N	N	N	N	2
	IQ: ID									
	Age: 7.3 ± 3.1									
	Male: 52.7%									
	Topic: Prolonged toilet training in children with DS									
	Country: Brazil									
4. Chandler et al. (2013)	*n*: 295	Cross-sectional study	Clayden et al., 2005	Y	Y	N	N	Y	N	3
	IQ: mean 70									
	Age: range 10–14									
	Male: not specified									
	Topic: Parent-reported gastro-intestinal symptoms in children with autism spectrum disorders to controls									
	Country: United Kingdom									
5. Connor et al. (2014)	*n*: 181	Randomised controlled trial intervention study	Castledine et al., 2013, Collins and Burch, 2009, Joanna Briggs Institute, 2008; Böhmer et al., 2001; Prasher & Smith, 2002	Y	Y	N	N	Y	N	3
	IQ: ID									
	Age: adults									
	Male: not specified									
	Topic: Using abdominal massage to manage constipation in people with learning disabilities									
	Country: United Kingdom									
6. Del Giudice et al. (1999)	*n*: 58	Cross-sectional study	Corazziari et al., 1985	Y	Y	N	N	Y	N	3
	IQ: range 20–50									
	Age: 0.6–12 years									
	Male: 43.1%									
	Topic: Prevalence and nature of gastrointestinal (GI) symptoms in children affected by cerebral palsy referred to a pediatric neurology outpatient clinic									
	Country: Italy									
7. Giesbers et al. (2012)	*n*: 70	Comparative study	Rasquin et al., 2006	Y	Y	N	N	N	N	2
	IQ: <20									
	Age: range 5.3–47.3									
	Male: 0%									
	Topic: Incontinence in individuals with Rett syndrome with profound ID Country: The Netherlands									
8. Hermans and Evenhuis (2014)	*n*: 1047	Cross-sectional study	No reference to a source	N	Uc	N	N	N	N	0
	IQ: range 70 - <20									
	Age: ≥50									
	Male: 51.3%									
	Topic: Multimorbidity in older adults with ID									
	Country: The Netherlands									
9. Jauhari et al. (2012)	*n:* 122	Prospective study	Veugelers et al., 2010	Y	Uc	Y	N	N	N	2
	IQ: <70									
	Age: range 0.6–13.3									
	Male: 68.8%									
	Topic: Comorbidities associated with intellectual disability among pediatric outpatients									
	Country: India									
10. Kinnear et al. (2018)	*n:* 1023	Cross-sectional study	ICD 10	Y	Y	N	N	N	N	2
	IQ: range 70- <20									
	Age: mean 43.9 (16–83)									
	Male: 54.9%									
	Topic: Prevalence of physical conditions and multimorbidity in a cohort of adults with ID with and without DS									
	Country: Scotland									
11. Larson et al. (2015)	*n*: 110	Cross-sectional study	No reference to a source	N	Uc	N	N	N	N	0
	IQ: - ID									
	Age: mean 24, range 16–50									
	Male: 50%									
	Topic: Investigate the primary health issues affecting adults with Angelman Syndrome									
	Country: England									
12. Maki et al. (2018)	*n*: 38	Double-blind study	Bristol Stool Scale	Y	Uc	Y	N	N	N	2
	IQ: ID									
	Age: not specified									
	Male: not specified									
	Topic: Probiotic for preventing constipation among people with ID									
	Country: Japan									
13. Marsh and Sweeney (2008)	*n*: 1	Case study	Benninga et al., 2004, Folden, 2002, Kyle et al., 2006	Y	Y	Y	N	N	N	3
	IQ: between 49-35									
	Age: 24									
	Male: 0									
	Topic: Non-pharmacological treatment of constipation people with ID									
	Country: Ireland									
14. Morad et al. (2007)	*n*: 2283	Cross-sectional study	Janicki et al., 1999, Janicki et al., 2002	Y	Y	Y	N	Y	N	4
	IQ: <50									
	Age: mean 49.8									
	Male: 51.4%									
	Topic: Prevalence and risk factors of constipation in residential care centers for adults with ID									
	Country: Israel									
15. Moss et al. (2008)	*n*: 5	Single case study design	Croffie et al., 2000, Gordon et al., 2002	Y	Y	Y	N	N	N	3
	IQ: severe and profound learning difficulties									
	Age: 4–9, mean 6.2									
	Male: 20%									
	Topic: Abdominal massage for the treatment of idiopathic constipation in children with profound learning disabilities									
	Country: England									
16. Murata et al. (2017)	*n:* 27	Retrospective study	Lewis & Heaton, 1997; Longstreth et al., 2006	Y	Uc	Y	N	N	N	2
	IQ: 35									
	Age: 2-45									
	Male: 70.3%									
	Topic: Determine the correlation between constipation and carnitine of patients with severe motor and intellectual disabilities									
	Country: Japan									
17. Niemczyk et al., 2016	*n:* 22 FXS/22 controls	Cross-sectional study	Rasquin et al., 2006	Y	Uc	N	N	N	N	1
	IQ: mean 70.9									
	Age: mean 11.0									
	Male: 100%									
	Topic: Incontinence and psychological problems in children with FXS in their home environments.									
	Country: Germany									
18. Smith et al. (2009)	*n*: 51 ASD/35 special school/112 regular school	A case control study	No reference to a source.	N	N	Uc	N	N	N	0
	IQ: special school <75									
	Age: ASD mean 9.7 special school mean 12.58 regular school mean 10.0									
	Male: not specified									
	Topic: Bowel symptom questionnaire compared children with ASD with control groups									
	Country: United Kingdom									
19. Staiano et al. (2000)	*n*: 20	Double-blind study	Staiano et al., 1991, Staiano et al., 1994, Staiano et al., 1996	Y	Y	Y	N	Y	N	4
	IQ: < 35									
	Age: not specified									
	Male: 70.0%									
	Topic: Effect of the dietary fiber glucomannan on chronic constipation in neurologically impaired children									
	Country: Italy									
20. Tse et al. (2000)	*n*: 20	Cross-sectional study	Corazziari et al., 1985	Y	Y	N	N	Y	N	3
	IQ: Severe developmental disabilities									
	Age: Range 3 - 17									
	Male: Not specified									
	Topic: Dietary fibre intake and constipation in children with severe developmental disabilities									
	Country: China									
21. Van Everdingen-Faasen et al. (2008)	*n*: 141	Cross-sectional study	Loening-Baucke, 1990, Rasquin-Weber et al., 1999	Y	Y	Y	N	N	N	3
	IQ: 70-40									
	Age: mean age 9.6 (range 6.5–16.5)									
	Male: 72.3%									
	Topic: Psychosocial co-morbidity affects treatment outcome in children with fecal incontinence									
	Country: Netherlands									
22. Van Winckel et al. (1999)	*n:* 420	Descriptive cross-sectional study	Whitehead and Dahlgren, 1991	Y	Y	N	N	Y	N	3
	IQ: range 50-<20									
	Age: 72									
	Male: 62.6%									
	Topic: Use of laxatives in institutions for the mentally retarded									
	Country: Belgium									
23. Vande Velde et al. (2010)	*n:* 58	Cross-sectional study	Böhmer et al., 2001; Longstreth et al., 2006; Harari et al., 1996	Y	Y	Y	Y	Y	N	5
	IQ: range 50 -<20									
	Age: range 27–41									
	Male: 50.0%									
	Topic: Measurements of colonic transit time in people with ID differentiates between retentive and non-retentive constipation.									
	Country: Belgium									
24. Veugelers et al. (2010)	*n*: 152	Cross-sectional observational study	Benninga et al., 2005, Rasquin et al., 2006; Böhmer et al., 2001	Y	Y	Y	Uc	Y	N	4
	IQ: range 34 -<20									
	Age: range 2–18									
	Male: 53.3%									
	Topic: Prevalence and clinical presentation of constipation in children with severe generalized CP and ID									
	Country: Netherlands									

Note: ASD = autism spectrum disorders, CAT = critical appraisal of topic, CP = cerebral palsy, DS = Down Syndrome, FXS = Fragile X Syndrome, n = Number of participants, N = no, ID = Intellectual Disabilities, IQ = Intelligence Quotient in points, Y = yes, Uc = Unclear.

As also shown in Table 1, the CAT scores of the included studies ranged between zero and six. In addition, to define constipation in people with (severe or profound) intellectual disabilities, six studies used standard definitions such as the Rome criteria (Giesbers et al., 2012; de Carvalho Mrad et al., 2014, 2018; Niemczyk et al., 2016), Bristol Stool Scale (Maki et al., 2018), and ICD 10 (Kinnear et al., 2018); four studies used separate signs and symptoms from another reference (Chandler et al.; Del Giudice et al., 1999; Jauhari et al., 2012; Van Winckel et al., 1999); and eleven studies used a self-composed definition (Böhmer et al., 2001; Connor et al., 2014; Marsh and Sweeney, 2008; Morad et al., 2007; Moss et al., 2008; Murata et al., 2017; Staiano et al., 2000; Van Everdingen-Faasen et al., 2008; Van de Velde et al., 2010; Veugelers et al., 2010; Tse et al., 2000). The other three studies gave no reference to a source of the definition Hermans and Evenhuis (2014); Larson et al. (2015); Smith et al. (2009).

‘Table 2 shows the symptoms and criteria related to existing definitions of obstipation. These symptoms and criteria differed in nature, as well as in how they could be determined. Possible ways to determine signs or symptoms were, for example, by observation, medical examination, or based on individual care and support plans or medical records such as medication use.’

**Table 2:** Overview of signs and symptoms found in the current review, related to previously described definitions.

Symptom	Criteria				
	Rome IV ∗	ICD-10 #	Bristol Stool Scale ˆ	Veugelers ∼	Other
Bowel movement <3 times a week	∗	#		∼	
Consistency dry hard stool	∗	#	ˆ	∼	
Difficult passage of stool		#	ˆ		
Form scybala stool				∼	
(Sensation of) incomplete evacuation	∗	#			
Straining during at least 25% of defections	∗				
Sensation of anorectal obstruction	∗				
Quantity					-
Delay in defecation		#			
Infrequent bowel movement		#			
Soiling					-
Stool withholding					-
Abdominal fullness				∼	
Bloating		#		∼	
Vomiting					-
Pain (abdominal/defecation)		#		∼	
Laxantia use					-
Abdominal examination for scybala				∼	
Measurement of colon transit time			ˆ	∼	
Manual disimpaction of feces				∼	
Presence of scybala by radiography/X-Ray				∼	
Presence of scybala by rectal exam					-

Note: ∗ = Rome IV Diagnostic Criteria (Hertz et al., 1996; Longstreth et al., 2006).

# = ICD-10 criteria.

ˆ = Criteria of the Bristol Stool Scale (Fallon et al., 2008, Lewis and Heaton, 1997).

∼ = Criteria of the proposed definition of Veugelers et al. (2010).

- = other.

Table 3 provides an overview of the signs and symptoms related to the CAT scores. The authors' definitions are organized first with the highest scoring definitions on the CAT on the left side of the table and the lowest scoring definitions on the right side. The signs and symptoms used most frequent are displayed at the top of the table with the least utilized at the bottom. The mostly used symptoms and signs in studies that scored three or higher on the CAT are: bowel movement <3 times a week (10); dry, hard stool, i.e. consistency (8); use of laxantia (6); difficult passage of stool (4); quantity, delay in defecation, infrequent bowel movement, pain (abdominal/defecation), and soiling (3). The five most used signs and symptoms of all of the studies without taking CAT scores into account were: bowel movement <3 times a week (16); dry, hard stool, i.e. consistency (14); difficult passage of stool (11); scybala stool, i.e. form (9); and use of laxantia (9). The least used signs and symptoms (1) that were described in the studies that scored a three or higher on the CAT: abdominal fullness, bloating, manual disimpaction of feces; presence of scybala as examined by radiography/X-Ray; presence of scybala as examined by rectal exam; stool withholding; and vomiting.

**Table 3:**
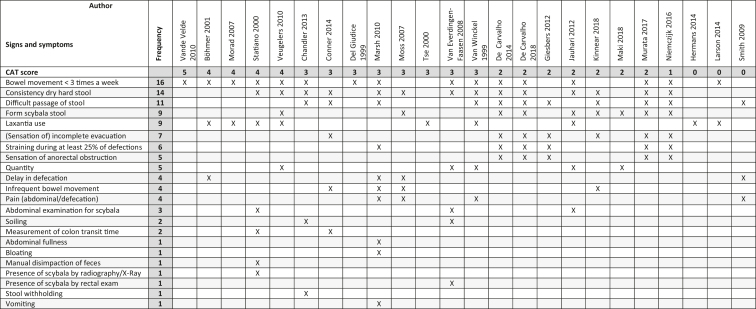
Signs and symptoms found in the articles in descending order (highest to lowest frequency score); columns ordered from left to right according to CAT scores.

Note: CAT = Checklist for text and opinion critical appraisal of topic.

## 4 Discussion

The aim of this review was to identify definitions of constipation and to analyse the signs and symptoms of constipation of people with severe or profound intellectual disabilities. A total of 24 studies were found related to people with intellectual disabilities in which a broad range of signs and symptoms were described, and whereby the definitions showed differences in quality scores. Only a limited number of the studies identified constipation in people with intellectual disabilities using the Rome criteria, ICD 10, and Bristol Stool Scale. These standard criteria are operational for people without an intellectual disability; however, their one-on-one use is questionable for people with severe or profound intellectual disabilities. Many of the included studies employed a self-composed definition, taking into account the specific characteristics of the target group of persons with (severe or profound) intellectual disabilities. However, these self-composed definitions had not been evaluated after being used for the target group, and no scientific substantiation was available.

An overview of studies that scored high on the CAT and the signs and symptoms used by the authors was provided. When examining the studies that have a score of three or higher on the CAT, it should be noted that the diversity in signs and symptoms of these definitions is higher than in the studies that scored lower on the CAT. In addition, the signs and symptoms described in the standardized lists of the Rome criteria, Bristol Stool Scale, and ICD 10 are not or are ambiguously described within the higher scored definitions. Future research should further explore the relation of the signs and symptoms we found with the standardized lists.

The approach used in the current study was to identify definitions for constipation in people with intellectual disabilities in literature, examine their quality, and explore their signs and symptoms. In order to be able to assess how the definitions that are currently being used were created, they were assessed on the basis of an adapted version of CAT (McArthur et al., 2015). The CAT criteria provided insight into the quality of the definitions of constipation in people with intellectual disabilities. Self-composed definitions adapted for the target population revealed higher scores on the CAT than standardized definitions such as the Rome criteria, the Bristol Stool Scale, or ICD 10. The definition used by (Vande Velde et al., 2010) scored high on the CAT; however, this author only used one item to define constipation in people with severe or profound intellectual disabilities, ‘Bowel movement <3 times a week’. In addition to this, no studies scored ‘yes’ on criterion 6 ‘Incongruence defended’, indicating that the definition used was substantiated.

In the majority of studies, the item frequency was used as predictive for constipation, for example, according to the Rome criteria. However, recently conducted research has shown that difficulty during relieving is more predictive for constipation than frequency (Enders, 2015). In addition, children with constipation showed a higher frequency of defection than children without constipation (Gulati et al., 2018). Bowel movement <3 times a week may therefore provide no accurate information and hence, frequency is a symptom that should be further examined, also with regard to the deviation of a defecation pattern (Enders, 2015). In addition, in study books for nurses, signs and symptoms, such as odor, were described that might be used as a symptom for constipation in people with severe or profound intellectual disabilities but were not described in the included studies (Bulechek et al., 2016). Since this systematic review did not provide a substantiated definition of constipation for people with severe or profound intellectual disabilities, such additional findings could be of great importance for identifying signs and symptoms to be used in future research when working further towards a definition of constipation in this group of people.

### 4.1 Strengths and limitations of the review

The authors used the CAT (McArthur et al., 2015) to assess the quality of the definitions which is a strength because this enabled demonstrating which signs and symptoms may be more suitable to define constipation in persons with (severe or profound) intellectual disabilities. However, to be able to employ this CAT for the quality of the definitions, the items of the CAT have been amended. Although these adjustments were made in consultation by all of the authors in this current study, this may have caused a bias. However, because there is no suitable instrument to only assess the quality of a definition, the adapted CAT seemed the best available instrument that could be used.

Combining the frequency of signs and symptoms with the quality assessment enabled visualizing signs and symptoms that are probably more suitable to define constipation in persons with severe or profound intellectual disabilities, which is a strength. The self-composed definitions are more focused on the observable signs and symptoms of constipation and make almost no use of questions that have to be answered by the person in question. This is an important prerequisite because people with a severe or profound intellectual disability are often not able to answer these questions in a manner that the people supporting them can understand (Coleman and Spurling, 2010). However, further research is needed into the signs of symptoms found in the present study, in order to take the next step toward a definition of constipation in people with severe or profound intellectual disabilities.

## 5 Conclusion

From the studies found with the systematic review, no definition emerged that is specifically substantiated for people with severe or profound intellectual disabilities. Results showed a wide variety between the definitions that were employed, ranging from standard definitions to self-composed definitions specifically for the target group. The definitions formulated specifically for people with severe or profound intellectual disabilities scored highest on the CAT; however, these definitions have not been tested and substantiated.

In the included studies, many different signs and symptoms were described that may indicate constipation. The aim of this review was to identify definitions of constipation and to analyse the signs and symptoms of constipation of people with severe or profound intellectual disabilities. However, the signs and symptoms we found, may not all be applicable as indicator of constipation in people with severe or profound intellectual disabilities, because they are not able to express some of the signs and symptoms found. In addition, part of the criteria from existing definitions cannot easily be answered by their family or professional caregivers. Personal and professional experiences of family members and direct support persons, nurses, and physicians for persons with intellectual disabilities may be useful to operationalize the signs and symptoms found in this literature review. Therefore, further research will be necessary to explore the described signs and symptoms with experts supporting people with severe or profound intellectual disabilities, when working further towards a definition of constipation in this group of people.

## Declarations

### Author contribution statement

Marjolijn C. Wagenaar: Conceived and designed the experiments; Performed the experiments; Analyzed and interpreted the data; Contributed reagents, materials, analysis tools or data; Wrote the paper.

Annette A. J. van der Putten and Cees P. van der Schans: Conceived and designed the experiments; Analyzed and interpreted the data; Wrote the paper.

Johanna G. Douma: Analyzed and interpreted the data; Contributed reagents, materials, analysis tools or data; Wrote the paper.

Aly Waninge: Conceived and designed the experiments; Analyzed and interpreted the data; Contributed reagents, materials, analysis tools or data; Wrote the paper.

### Funding statement

This research did not receive any specific grant from funding agencies in the public, commercial, or not-for-profit sectors.

### Data availability statement

Data included in article/supplementary material/referenced in article.

### Declaration of interests statement

The authors declare no conflict of interest.

### Additional information

No additional information is available for this paper.

## Figures and Tables

**Table A:** Critical Appraisal of Topic (CAT) for Text and Opinion papers (McArthur et al., 2015) adapted for the present study.

	Yes	No	Unclear	Not applicable
1. Is the source of the definition that is used in the article clearly identified by the authors?				
2. Does at least one of the authors of the article have standing in the field of expertise∗ (severe or profound) intellectual disabilities?				
3. Are the interests of the relevant population (severe or profound) intellectual disabilities the central focus of the definition in the source, or is it clear why this definition was chosen for participants with (severe or profound) intellectual disabilities in the current article?				
4. Is the stated definition the result of an analytical process, and is there logic in the argumentation expressed?				
5. Is there reference to the extant literature (more than 1 article) and is it a non-biased representation?				
6. Is any incongruence with the literature/sources logically defended, is the author aware of alternate or dominant definitions?				

∗ The author has expertise with the target group through work or by having written more than one scientific publication about the target group.

## Appendix A Search string for database searches.

### MEDLINE

((MH constipation OR MH "Fecal Impaction" OR MH laxatives OR TI (bowel OR cathartics OR colon∗ OR constipate∗ OR defecat∗ OR defaecat∗ OR fecal OR faecal OR laxative∗) OR AB (bowel OR cathartics OR colon∗ OR constipate∗ OR defecat∗ OR defaecat∗ OR fecal OR faecal OR laxative∗)) AND ((MH "Developmental Disabilities" OR MH "Intellectual Disability" OR MH "Mentally Disabled Persons" OR TI ( cognitiv∗ challen∗ OR cognitiv∗ deficien∗ OR cognitiv∗ deficit∗ OR cognitiv∗ disab∗ OR cognitiv∗ handicap∗ OR cognitiv∗ impair∗ OR cognitiv∗ retard∗ OR development∗ disab∗ OR development∗ disord∗ OR disabilit∗ disord∗ OR intellectual∗ deficien∗ OR intellectual∗ deficit∗ OR intellectual∗ disab∗ OR intellectual∗ disord∗ OR intellectual∗ handicap∗ OR intellectual∗ impair∗ OR intellectual∗ retard∗ OR mental∗ challen∗ OR mental∗ deficien∗ OR mental∗ disab∗ OR mental∗ disord∗ OR mental∗ handicap∗ OR mental∗ impair∗ OR mental∗ retard∗ OR profound multiple disabilit∗ OR severe multiple disabilit∗) OR AB (cognitiv∗ challen∗ OR cognitiv∗ deficien∗ OR cognitiv∗ deficit∗ OR cognitiv∗ disab∗ OR cognitiv∗ handicap∗ OR cognitiv∗ impair∗ OR cognitiv∗ retard∗ OR development∗ disab∗ OR development∗ disord∗ OR disabilit∗ disord∗ OR intellectual∗ deficien∗ OR intellectual∗ deficit∗ OR intellectual∗ disab∗ OR intellectual∗ disord∗ OR intellectual∗ handicap∗ OR intellectual∗ impair∗ OR intellectual∗ retard∗ OR mental∗ challen∗ OR mental∗ deficien∗ OR mental∗ disab∗ OR mental∗ disord∗ OR mental∗ handicap∗ OR mental∗ impair∗ OR mental∗ retard∗ OR profound multiple disabilit∗ OR severe multiple disabilit∗))

### Embase

('constipation'/exp OR 'defecation disorder'/exp OR 'laxative'/exp OR Bowel:ti,ab OR Cathartics:ti,ab OR Colon∗:ti,ab OR Constip∗:ti,ab OR Defaecat∗:ti,ab OR Defecat∗:ti,ab OR Faecal:ti,ab OR Fecal:ti,ab OR Laxat∗:ti,ab) AND ('developmental disorder'/exp OR 'mentally disabled person'/exp OR 'mental deficiency'/exp OR ‘Cognitiv∗ challen∗’:ti,ab OR ‘Cognitiv∗ deficien∗’:ti,ab OR ‘Cognitiv∗ deficit∗’:ti,ab OR ‘Cognitiv∗ disab∗’:ti,ab OR ‘Cognitiv∗ handicap∗’:ti,ab OR ‘Cognitiv∗ impair∗’:ti,ab OR ‘Cognitiv∗ retard∗’:ti,ab OR ‘Developmental∗ disab∗’:ti,ab OR ‘Developmental∗ disord∗’:ti,ab OR ‘Disabilit∗ disorder∗’:ti,ab OR ‘Intellectual∗ deficien∗’:ti,ab OR ‘Intellectual∗ deficit∗’:ti,ab OR ‘Intellectual∗ disab∗’:ti,ab OR ‘Intellectual∗ disord∗’:ti,ab OR ‘Intellectual∗ handicap∗’:ti,ab OR ‘Intellectual∗ impair∗’:ti,ab OR ‘Intellectual∗ retard∗’:ti,ab OR ‘Mental∗ deficien∗’:ti,ab OR ‘Mental∗ disab∗’:ti,ab OR ‘Mental∗ disord∗’:ti,ab OR ‘Mental∗ handicap∗’:ti,ab OR ‘Mental∗ impair∗’:ti,ab OR ‘Mental∗ retard∗’:ti,ab OR ‘Mental∗ challen∗’:ti,ab OR ‘Profound multiple disabilit∗’:ti,ab OR ‘Severe multiple disabilit∗’:ti,ab)

### Cochrane CENTRAL

(bowel OR cathartics OR colon∗ OR constipate∗ OR defecat∗ OR defaecat∗ OR fecal OR faecal OR laxative∗) AND (cognitiv∗ challen∗ OR cognitiv∗ deficien∗ OR cognitiv∗ deficit∗ OR cognitiv∗ disab∗ OR cognitiv∗ handicap∗ OR cognitiv∗ impair∗ OR cognitiv∗ retard∗ OR development∗ disab∗ OR development∗ disord∗ OR disabilit∗ disord∗ OR intellectual∗ deficien∗ OR intellectual∗ deficit∗ OR intellectual∗ disab∗ OR intellectual∗ disord∗ OR intellectual∗ handicap∗ OR intellectual∗ impair∗ OR intellectual∗ retard∗ OR mental∗ challen∗ OR mental∗ deficien∗ OR mental∗ disab∗ OR mental∗ disord∗ OR mental∗ handicap∗ OR mental∗ impair∗ OR mental∗ retard∗ OR profound multiple disabilit∗ OR severe multiple disabilit∗)

### CINAHL

((MH constipation OR MH "Feces, Impacted" OR MH Cathartics OR TI ( bowel OR cathartics OR colon∗ OR constipate∗ OR defecat∗ OR defaecat∗ OR fecal OR faecal OR laxative∗) OR AB (bowel OR cathartics OR colon∗ OR constipate∗ OR defecat∗ OR defaecat∗ OR fecal OR faecal OR laxative∗)) AND ((MH "Developmental Disabilities" OR MH "Intellectual Disability" OR MH "Mentally Disabled Persons" OR TI ( cognitiv∗ challen∗ OR cognitiv∗ deficien∗ OR cognitiv∗ deficit∗ OR cognitiv∗ disab∗ OR cognitiv∗ handicap∗ OR cognitiv∗ impair∗ OR cognitiv∗ retard∗ OR development∗ disab∗ OR development∗ disord∗ OR disabilit∗ disord∗ OR intellectual∗ deficien∗ OR intellectual∗ deficit∗ OR intellectual∗ disab∗ OR intellectual∗ disord∗ OR intellectual∗ handicap∗ OR intellectual∗ impair∗ OR intellectual∗ retard∗ OR mental∗ challen∗ OR mental∗ deficien∗ OR mental∗ disab∗ OR mental∗ disord∗ OR mental∗ handicap∗ OR mental∗ impair∗ OR mental∗ retard∗ OR profound multiple disabilit∗ OR severe multiple disabilit∗) OR AB (cognitiv∗ challen∗ OR cognitiv∗ deficien∗ OR cognitiv∗ deficit∗ OR cognitiv∗ disab∗ OR cognitiv∗ handicap∗ OR cognitiv∗ impair∗ OR cognitiv∗ retard∗ OR development∗ disab∗ OR development∗ disord∗ OR disabilit∗ disord∗ OR intellectual∗ deficien∗ OR intellectual∗ deficit∗ OR intellectual∗ disab∗ OR intellectual∗ disord∗ OR intellectual∗ handicap∗ OR intellectual∗ impair∗ OR intellectual∗ retard∗ OR mental∗ challen∗ OR mental∗ deficien∗ OR mental∗ disab∗ OR mental∗ disord∗ OR mental∗ handicap∗ OR mental∗ impair∗ OR mental∗ retard∗ OR profound multiple disabilit∗ OR severe multiple disabilit∗))

### PsycINFO

((DE "Constipation" OR DE “cathartics” OR TI ( bowel OR cathartics OR colon∗ OR constipate∗ OR defecat∗ OR defaecat∗ OR fecal OR faecal OR laxative∗) OR AB (bowel OR cathartics OR colon∗ OR constipate∗ OR defecat∗ OR defaecat∗ OR fecal OR faecal OR laxative∗)) AND ((DE "Developmental Disabilities" OR DE "Intellectual Development Disorder" OR DE "Cognitive Impairment" OR DE "Mental Disorders" OR TI ( cognitiv∗ challen∗ OR cognitiv∗ deficien∗ OR cognitiv∗ deficit∗ OR cognitiv∗ disab∗ OR cognitiv∗ handicap∗ OR cognitiv∗ impair∗ OR cognitiv∗ retard∗ OR development∗ disab∗ OR development∗ disord∗ OR disabilit∗ disord∗ OR intellectual∗ deficien∗ OR intellectual∗ deficit∗ OR intellectual∗ disab∗ OR intellectual∗ disord∗ OR intellectual∗ handicap∗ OR intellectual∗ impair∗ OR intellectual∗ retard∗ OR mental∗ challen∗ OR mental∗ deficien∗ OR mental∗ disab∗ OR mental∗ disord∗ OR mental∗ handicap∗ OR mental∗ impair∗ OR mental∗ retard∗ OR profound multiple disabilit∗ OR severe multiple disabilit∗) OR AB (cognitiv∗ challen∗ OR cognitiv∗ deficien∗ OR cognitiv∗ deficit∗ OR cognitiv∗ disab∗ OR cognitiv∗ handicap∗ OR cognitiv∗ impair∗ OR cognitiv∗ retard∗ OR development∗ disab∗ OR development∗ disord∗ OR disabilit∗ disord∗ OR intellectual∗ deficien∗ OR intellectual∗ deficit∗ OR intellectual∗ disab∗ OR intellectual∗ disord∗ OR intellectual∗ handicap∗ OR intellectual∗ impair∗ OR intellectual∗ retard∗ OR mental∗ challen∗ OR mental∗ deficien∗ OR mental∗ disab∗ OR mental∗ disord∗ OR mental∗ handicap∗ OR mental∗ impair∗ OR mental∗ retard∗ OR profound multiple disabilit∗ OR severe multiple disabilit∗))

## References

[bib73] Benninga M.A., Candy D.C., Taminiau J.A. (2005). New treatment options in childhood constipation?. J. Pediatr. Gastroenterol. Nutr..

[bib72] Benninga M.A., Voskuijl W.P., Taminiau J.A.J.M. (2004). Childhood Constipation: Is There New Light in The Tunnel?. J. Pediatr. Gastroenterol. Nutr..

[bib1] Bharucha A.E., Pemberton J.H., Locke G.R. (2013). American gastroenterological association technical review on constipation. Gastroenterol..

[bib2] Böhmer C.J., Taminiau J.A., Klinkenberg-Knol E., Meuwissen S.G. (2001). The prevalence of constipation in institutionalized people with intellectual disability. J. Intellect. Disabil. Res.: JIDR (J. Intellect. Disabil. Res.).

[bib3] Bragg C.L., Edwards-Beckett J., Eckle N., Principe K., Terry D. (1994). Shunt dysfunction and constipation: could there be a link?. J. Neurosci. Nurs.: J. Am. Assoc. Neurosci. Nurs..

[bib4] Bulechek G.M., Butcher H.K., Dochterman J.M., Wagner C.M., Bulechek G.M., Butcher H.K., Dochterman J.M., Wagner C.M. (2016). *Verpleegkundige Interventies.* (H. Merkus Trans.). (Fourth, Revised Edition.

[bib74] Castledine G., Grainger M., Wood N., Dilley C. (2013). Researching the management of constipation in long-term care: Part 1. Br. J. Nurs..

[bib5] Chandler S., Carcani-Rathwell I., Charman T., Pickles A., Loucas T., Meldrum D., Simonoff E., Sullivan P., Baird G. (2013). Parent-reported gastro-intestinal symptoms in children with autism spectrum disorders. J. Autism Dev. Disord..

[bib50] Clayden G.S., Keshtgar A.S., Carcani-Rathwell I. (2005). The management of chronic constipation and related faecal incontinence in childhood. Arch Dis Child Educ Pract Ed.

[bib6] Coleman J., Spurling G. (2010). Constipation in people with learning disability. BMJ.

[bib76] Collins B., Burch J. (2009). Constipation, treatment and biofeedback therapy. Br J Community Nurs.

[bib7] Connor M., Hunt C., Lindley A., Adams J. (2014). Using abdominal massage in bowel management. Nurs. Stand..

[bib52] Corazziari E., Cucchiara S., Staiano A, Romaniello G., Tamburrini O., Torsoli A., Auricchio S. (1985). Bowel function in constipation. Gastrointestinal transit time, frequency of defecation, and anorectal manometry in healthy and constipated children. J Pediatr.

[bib53] Croffie J.M., Fitzgerald J.F., Chong K.F. (2000). Recurrent abdominal pain in children-a retrospective study of outcome in a group referred to a pediatric gastroenterology practice. Clin. Pediatr..

[bib8] Cvetković D., Živković V., Damjanjuk I., Nikolić S. (2019). Intestinal obstruction as a cause of death in the mentally disabled. Forensic Sci. Med. Pathol..

[bib9] de Carvalho Mrad F.C., de Bessa J., de Rezende Duarte A.M.B., Vieira A.A.P., Araujo F.C.C., de Sá Camargo M.L., Tibiriça S.H.M., de Figueiredo A.A., de Bastos Netto J.M. (2014). Prevalence of lower urinary tract symptoms in individuals with Down syndrome. J. Pediatr. Urol..

[bib10] de Carvalho Mrad F.C., deFigueiredo A.A., de Bessa J., de Bastos Netto J.M. (2018). Prolonged toilet training in children with down syndrome: a case–control study. J. Pediatr..

[bib11] Del Giudice E., Staiano A., Capano G., Romano A., Florimonte L., Miele E., Ciarla C., Campanozzi A., Crisanti A.F. (1999). Gastrointestinal manifestations in children with cerebral palsy. Brain Dev..

[bib12] Enders G. (2015).

[bib54] Fallon A., Westaway J., Moloney C. (2008). A systematic review of psychometric evidence and expert opinion regarding the assessment of faecal incontinence in older community-dwelling adults. Int. J. Evid. Healthcare.

[bib55] Folden S.L. (2002). Practice guidelines for the management of constipation in adults rehabilitation. Nurs..

[bib13] Giesbers S., Didden R., Radstaake M., Korzilius H., Von Gontard A., Lang R., Smeets L.M.C., Curfs L.M.G. (2012). Incontinence in individuals with rett syndrome: a comparative study. J. Dev. Phys. Disabil..

[bib77] Gordon J., Reid P., Thompson C., Watford C. (2002). Idiopathic constipation management pathway. Nursing Times.

[bib14] Gulati R., Komuravelly A., Leb S., Mhanna M.J., Ghori A., Leon J., Needlman R. (2018). Usefulness of assessment of stool from by the modified Bristol stool from scale in primary care pediatrics. Pediatr. Gastroenterol. Hepatol. Nutr..

[bib60] Harari D., Gurwitz J.H., Avorn J., Bohn R., Minaker K.L. (1996). Bowel habit in relation to age and gender. Findings from the National Health Interview Survey and clinical implications. Arch Intern Med.

[bib15] Hermans H., Evenhuis H.M. (2014). Multimorbidity in older adults with intellectual disabilities. Res. Dev. Disabil..

[bib16] Herz M.J., Kahan E., Zalevski S., Aframian R., Kuznitz D., Reichman S. (1996). Constipation: a different entity for patients and doctors. Fam. Pract..

[bib17] Jancar J., Speller C.J. (1994). Fatal intestinal obstruction in the mentally handicapped. J. Intellect. Disabil. Res..

[bib59] Janicki M.P., Dalton A.J., Henderson C.M., Davidson P.W. (1999). Mortality and morbidity among older adults with intellectual disability: health services considerations. Disabil. Res.

[bib57] Janicki M.P., Davidson P.W., Henderson C.M., McCallion P., Taets J.D., Force L.T., Sulkes E. Frangenberg S.B., Ladrigan P.M. (2002). Health characteristics and health services utilization in older adults with intellectual disability living in community residences. J. Intellect. Disabil. Res..

[bib18] Jauhari P., Bhargava R., Bhave A., Kumar C., Kumar R. (2012). Comorbidities associated with intellectual disability among pediatric outpatients seen at a teaching hospital in northern India. J. Pol. Pract. Intellect. Disabil..

[bib78] Joanna Briggs Institute (2008). https://www.joannabriggs.edu.au/documents/JBIReviewManual_CiP11449.pdf.

[bib19] Kinnear D., Morrison J., Allan L., Henderson A., Smiley E., Cooper S.A. (2018). Prevalence of physical conditions and multimorbidity in a cohort of adults with intellectual disabilities with and without down syndrome: cross-sectional study. BMJ Open.

[bib20] Koppen I.J., Nurko S., Saps M., Di Lorenzo C., Benninga M.A. (2017). The pediatric Rome IV criteria: what’s new?. Expet Rev. Gastroenterol. Hepatol..

[bib63] Kyle U.G., Kossovsky M.P., Karsegard V.L., Pichard C. (2006). Comparison of tools for nutritional assessment and screening at hospital admission: a population study. Clin Nutr.

[bib21] Larson A.M., Shinnick J.E., Shaaya E.A., Thiele E.A., Thibert R.L. (2015). Angelman syndrome in adulthood. Am. J. Med. Genet..

[bib81] Lewis S.J., Heaton K.W. (1997). Stool form scale as a useful guide to intestinal transit time. Scandinavian J Gastroenterol.

[bib82] Loening-Baucke V. (1990). Biofeedback therapy for fecal incontinence. Dig Dis (Basel, Switzerland).

[bib22] Longstreth G.F., Thompson W.G., Chey W.D., Houghton L.A., Mearin F., Spiller R.C. (2006). Functional bowel disorders. Gastroenterology.

[bib24] Maki R., Matsukawa M., Matsuduka A., Hashinaga M., Anai H., Yamaoka Y., Hanada K., Fujii C. (2018). Therapeutic effect of lyophilized, Kefir-fermented milk on constipation among persons with mental and physical disabilities. Jpn. J. Nurs. Sci..

[bib25] Marsh L., Sweeney J. (2008). Nurses' knowledge of constipation in people with learning disabilities. Br. J. Nurs..

[bib26] McArthur A., Klugárová J., Yan H., Florescu S. (2015). Innovations in the systematic review of text and opinion. Int. J. Evid. Base. Healthc..

[bib27] McCarron M., Swinburne J., Burke E., McGlinchey E., Carroll R., McCallion P. (2013). Patterns of multimorbidity in an older population of persons with an intellectual disability: results from the intellectual disability supplement to the Irish longitudinal study on aging (IDS-TILDA). Res. Dev. Disabil..

[bib28] Moher D., Liberati A., Tetzlaff J., Altman D.G. (2009). Preferred reporting items for systematic reviews and meta-analyses: the PRISMA statement. BMJ.

[bib29] Morad M., Nelson N.P., Merrick J., Davidson P.W., Carmeli E. (2007). Prevalence and risk factors of constipation in adults with intellectual disability in residential care centers in Israel. Res. Dev. Disabil..

[bib30] Moss L., Smith M., Wharton S., Hames A. (2008). Abdominal massage for the treatment of idiopathic constipation in children with profound learning disabilities: a single case study design. Br. J. Learn. Disabil..

[bib31] Murata S., Inoue K., Aomatsu T., Yoden A., Tamai H. (2017). Supplementation with carnitine reduces the severity of constipation: a retrospective study of patients with severe motor and intellectual disabilities. J. Clin. Biochem. Nutr..

[bib32] Nakken H., Vlaskamp C. (2007). A need for a taxonomy for profound intellectual and multiple disabilities. J. Pol. Pract. Intellect. Disabil..

[bib33] Niemczyk J., Von Gontard A., Equit M., Bauer K., Naumann T., Wagner C., Curfs L. (2016). Detailed assessment of incontinence in boys with fragile-X-syndrome in a home setting. Eur. J. Pediatr..

[bib34] Patja K., Mölsä P., Iivanainen M. (2001). Cause-specific mortality of people with intellectual disability in a population-based, 35-year follow-up study. J. Intellect. Disabil. Res..

[bib35] Peppas G., Alexiou V.G., Mourtzoukou E., Falagas M.E. (2008). Epidemiology of constipation in Europe and Oceania: a systematic review. BMC Gastroenterol..

[bib64] Prasher V.P., Smith B. (2002). Down Syndrome and Health Care.

[bib80] Rasquin A., Lorenzo C., di, Forbes D., Guiraldes E., Hyams J.S., Staiano A. (2006). Childhood functional gastrointestinal disorders: child/adolescent. Gastroenterol..

[bib79] Rasquin-Weber A., Hyman P.E., Cucchiara S., Fleisher D.R., Hyams J.S., Milla P.J. (1999). Childhood functional gastrointestinal disorders. Gut.

[bib36] Robertson J., Baines S., Emerson E., Hatton C. (2018). Prevalence of constipation in people with intellectual disability: a systematic review. J. Intellect. Dev. Disabil..

[bib37] Schalock R.L., Luckasson R., Tassé M.J. (2021).

[bib38] Smith R.A., Farnworth H., Wright B., Allgar V. (2009). Are there more bowel symptoms in children with autism compared to normal children and children with other developmental and neurological disorders? A case control study. Autism.

[bib39] Smout A.J.P.M. (2001).

[bib40] Sobrado C.W., Corrêa Neto I.J.F., Pinto R.A., Sobrado L.F., Nahas S.C., Cecconello I. (2018). Diagnosis and treatment of constipation: a clinical update based on the Rome IV criteria. J. Coloproctol. (Rio de Janeiro).

[bib68] Staiano A., Andreotti M.R., Greco L., Basile P., Auricchio S. (1994). Long-term follow-up of children with chronic idiopathic constipation. Dig. Dis. Sci..

[bib67] Staiano A., Cucchiara S., Andreotti M.R., Minella R., Manzi G. (1991). Effect of cisapride on chronic idiopathic constipation in children. Dig. Dis. Sci..

[bib69] Staiano A., Del Giudice E., Simeone D., Miele E., Marino A. (1996). Cisapride in neurologically impaired children with chronic constipation. Dig. Dis. Sci..

[bib41] Staiano A., Simeone D., Del Giudice E., Miele E., Tozzi A., Toraldo C. (2000). Effect of the dietary fiber glucomannan on chronic constipation in neurologically impaired children. J. Pediatr..

[bib42] Tse P.W.T., Leung S.S.F., Chan T., Sien A., Chan A.K.H. (2000). Dietary fibre intake and constipation in children with severe developmental disabilities. J. Paediatr. Child Health.

[bib43] Van Everdingen-Faasen E.Q., Gerritsen B.J., Mulder P.G.H., Fliers E.A., Groeneweg M. (2008). Psychosocial co-morbidity affects treatment outcome in children with fecal incontinence. Eur. J. Pediatr..

[bib44] Van Timmeren E.A., Van der Putten A.A.J., Van Schrojenstein Lantman-de Valk H.M.J., Van der Schans C.P., Waninge A. (2016). Prevalence of reported physical health problems in people with severe or profound intellectual and motor disabilities: a cross-sectional study of medical records and care plans. J. Intellect. Disabil. Res..

[bib45] Van Timmeren E.A., Van der Schans C.P., Van der Putten A.A.J., Krijnen W.P., Steenbergen H.A., Van Schrojenstein Lantman-de Valk H.M.J., Waninge A. (2017). Physical health issues in adults with severe or profound intellectual and motor disabilities: a systematic review of cross-sectional studies. J. Intellect. Disabil. Res..

[bib46] Van Winckel M., Vander Stichele R., De Bacquer D., Bogaert M. (1999). Use of laxatives in institutions for the mentally retarded. Eur. J. Clin. Pharmacol..

[bib47] Vande Velde S., Van Biervliet S., Van Goethem G., De Looze D., Van Winckel M. (2010). Colonic transit time in mentally retarded persons. Int. J. Colorectal Dis..

[bib48] Veugelers R., Benninga M.A., Calis E.A.C., Willemsen S.P., Evenhuis H., Tibboel D., Penning C. (2010). Prevalence and clinical presentation of constipation in children with severe generalized cerebral palsy. Dev. Med. Child Neurol..

[bib70] Whitehead M., Dahlgren G. (1991). What can be done about inequalities in health?. Lancet (London, England).

